# Hallux Flexion Deformity Secondary to Fibula Harvesting in a 10-Year-Old Patient With Neurofibromatosis

**DOI:** 10.7759/cureus.61850

**Published:** 2024-06-06

**Authors:** Freideriki Poutoglidou, Andrea Sott, Sohail Yousaf, Kunalan Maruthainar, Paul Hamilton

**Affiliations:** 1 Trauma and Orthopaedics, Epsom and St Helier University Hospitals NHS Trust/South West London Elective Orthopaedic Centre, Surrey, GBR

**Keywords:** fibula harvesting, joint contracture, flexion deformity, interphalangeal joint, hallux

## Abstract

Hallux interphalangeal joint (IPJ) flexion contracture is an uncommon deformity with various underlying causes, including trauma, neurological disorders, and connective tissue pathologies. We present a unique case of a 10-year-old female patient with neurofibromatosis type 1 (NF1) and a history of fibula transposition surgery, resulting in a hallux IPJ flexion contracture. We believe that the loss of the proximal fibular attachment of the extensor hallucis longus (EHL) following fibula harvesting resulted in EHL weakness and unopposed flexor hallucis longus (FHL) pull that eventually led to the contracture. The patient underwent various diagnostic assessments, ruling out other potential causes of the deformity. This case emphasizes the importance of considering previous surgical interventions when encountering flexion contractures of the toes.

## Introduction

Hallux interphalangeal joint (IPJ) flexion contracture represents a relatively uncommon deformity with diverse etiologies. Trauma-induced contractures, known as checkrein deformities, result from entrapment of the flexor hallucis longus (FHL) within fracture callus or fibrous scar tissue and have been documented in the literature [1-3]. Previous surgery on the foot or ankle can lead to scarring and contractures that can eventually cause toe deformities [2]. Neurological conditions characterized by lower limb spasticity, such as cerebral palsy or stroke, have been associated with IPJ contractures. These conditions can cause abnormal muscle tone and imbalances, leading to the persistent flexion of the hallux [4]. Plantar fibromatosis, commonly known as Ledderhose's disease, has been identified as another potential causative factor. This condition involves the thickening of the plantar fascia, which can indirectly affect the toe’s position and lead to contractures [5,6]. Furthermore, delayed or missed diagnosis of compartment syndrome may result in nerve injury or muscle necrosis leading to toe contractures due to the resultant ischemic damage and fibrosis [7].

Fibula harvesting presents another potential factor contributing to the development of a flexion contracture of the hallux. Apart from the observed hammer and claw toe deformities and deficits in the dorsal extension of the hallux, several other potential morbidities may arise. These include sensory disturbances such as numbness or tingling, which can result from injury to cutaneous nerves during the harvesting process. Additionally, alterations in ankle stability and gait patterns may occur, particularly in cases where weakness or dysfunction in the peroneal muscles is present. Furthermore, prolonged wound healing represents another potential donor site morbidity [8].

Effective management of hallux IPJ contractures remains challenging and depends on the underlying cause and severity. A comprehensive approach is required that includes either conservative measures or surgical interventions or both of them. Conservative treatment includes physiotherapy, orthotics, and splinting. When these measures are insufficient, surgical options such as soft tissue releases, tendon lengthening, or an IPJ fusion may be necessary to correct the deformity and restore function [9].

Here, we present a unique case of a 10-year-old female patient with a hallux IPJ flexion contracture in the setting of neurofibromatosis type 1 (NF1) and a history of fibula transposition surgery. To our knowledge, this represents the first reported case of its kind in the literature.

## Case presentation

**Figure 1:**
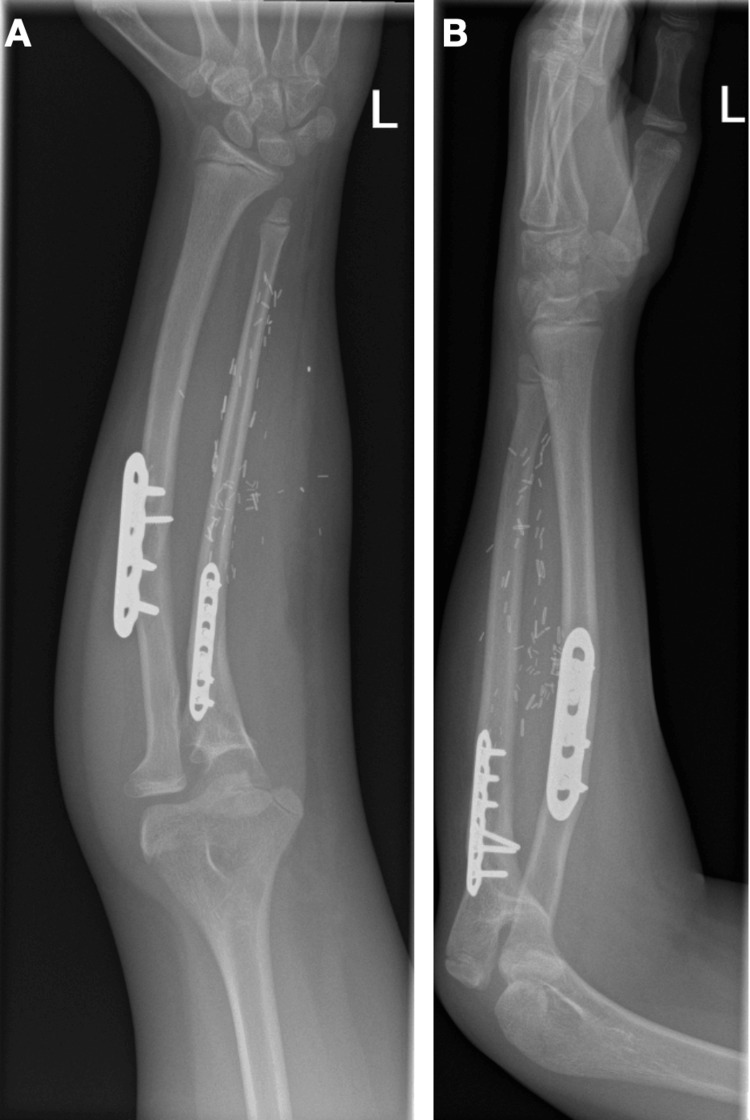
(A) AP and (B) lateral X-rays of the patient’s left forearm showing the transposed fibula to the left ulna AP, anteroposterior

A 10-year-old female patient was referred to the Elective Foot and Ankle Clinic of our department because of a hallux IPJ flexion contracture of her right foot that has developed over the last few years. No previous injuries were reported. The patient has a history of NF1 and she had a right fibula transposition to her left ulna four years ago for the management of ulnar pseudoarthrosis in Great Ormond Street Hospital in London (Figure 1). This was a single-stage procedure with no prior lengthening required. A lateral incision centered over the fibula was utilized for the harvesting procedure. At the time of surgery and during the early postoperative period, no donor site morbidity was observed. There were no other medical comorbidities.

Clinical examination revealed a mild flexible pes planus deformity bilaterally. The patient was able to raise double and single limb heel. There were no scars, skin breakdown, or signs of infection. Hallux passive IPJ flexion was from 10° extension to 40° flexion. The metatarsophalangeal joint (MPTJ) movement ranged from 20° plantarflexion to 70° dorsiflexion. There was no evidence of FHL tightness. The extensor hallucis longus (EHL) power was 4/5 on the right side and 5/5 on the left side. There were no sensory deficits. The patient had a normal gait, and the scoliosis examination was negative.

**Figure 2:**
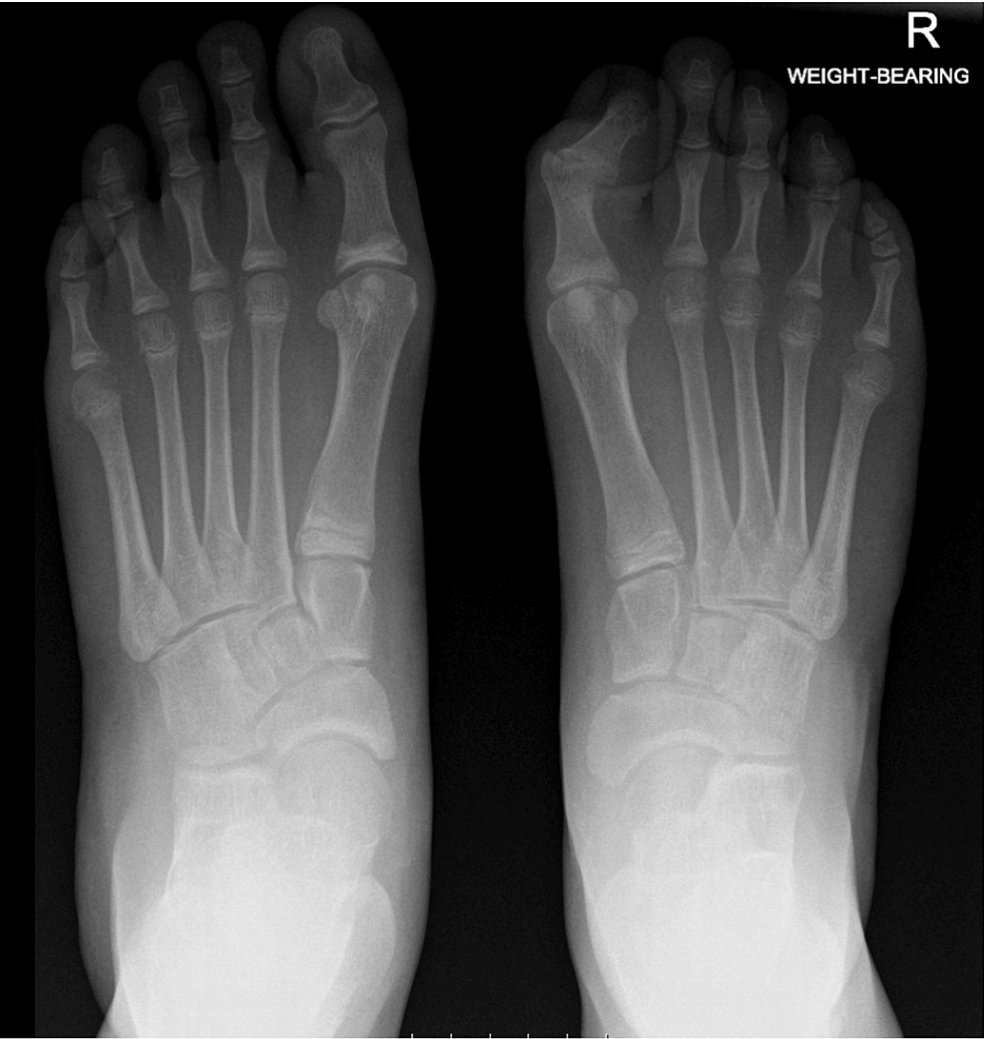
AP weight-bearing X-rays of both feet. The IPJ of the right hallux appears flexed and valgus angulated, but the X-rays are otherwise unremarkable AP, anteroposterior

**Figure 3:**
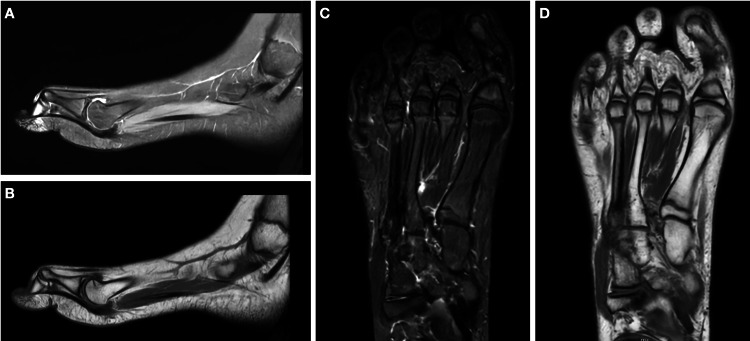
(A, B) Sagittal T2- and T1-weighted MRI scan images demonstrating a flexion of the IPJ of the right hallux with no other abnormal findings. (C, D) Axial T2- and T1-weighted MRI scan images demonstrating a flexion and valgus deviation of the IPJ of the right hallux with no other abnormal findings

Weight-bearing X-rays (Figure 2), an ultrasound scan, and an MRI scan (Figure 3) of the right foot were obtained. The investigations confirmed the presence of the deformity but were otherwise unremarkable. Subsequently, the patient underwent nerve conduction studies (NCS). The sensory studies showed symmetrical and normal sural and superficial peroneal nerves. The right medial plantar nerve was found to be slightly smaller than the left, but this had no clinical correlation. The motor peroneal studies were symmetrical and normal and the tibial CMAPs were also symmetrical and normal. Electromyography of the FHL demonstrated no abnormal activity.

The case was discussed in the complex case Foot and Ankle meeting of our department. Since no other probable cause was found, the hallux IPJ flexion contracture was attributed to the loss of the proximal fibular attachment of the EHL that resulted from the fibula harvesting four years ago. We believe that this resulted in the weakness of the EHL and the unopposed pull of the FHL eventually led to the flexion contraction of the hallux.

The patient is currently managed conservatively with physiotherapy and a hallux extension splint. An IPJ fusion has been discussed but both the patient and the parents would prefer to avoid surgery for the time being.

## Discussion

Flexion contracture of the hallux IPJ is a deformity with diverse etiologies, ranging from traumatic injuries to neurological disorders and connective tissue pathologies. In this case report, we present a unique scenario where a 10-year-old female patient with NF1 and a history of fibula transposition surgery developed a hallux IPJ flexion contracture.

NF1 is a genetic disorder caused by a mutation in the NF1 gene that codes for the neurofibromin protein and is characterized by neurofibromas, Lisch nodules, cafe-au-lait spots, and skeletal abnormalities. While NF1 primarily affects the nervous system, its musculoskeletal manifestations are well-documented, including anterolateral tibial bowing, radial or ulnar pseudoarthrosis, and scoliosis [10].

Free fibular grafts are commonly employed to address conditions such as congenital ulnar pseudoarthrosis or post-tumor resection bone defects [11]. EHL originates from the anterior surface of the mid-shaft of the fibula and the adjacent interosseous membrane. Depending on the extent of fibular resection, EHL’s proximal attachment can be disrupted leading to weakness of the EHL muscle and an unopposed pull of the FHL, eventually resulting in IPJ flexion contracture of the hallux. A previous biomechanical study on cadaveric lower legs has shown that free fibula flap surgery is associated with significant impairment of the EHL. On the other hand, extensor digitorum longus (EDL) was found not to be impaired significantly after fibula osteotomy and harvesting [12].

Shingade et al. found the isolated weakness of the EHL following harvesting of the fibula in 10 of 26 patients [13]. They performed an NCS that showed injury of the nerve to the EHL. In their study, almost all of the cases resolved spontaneously and NCS were normal three months later. Contracture of the hallux was not reported in any of their cases. A recently published study by Wu et al. investigated the incidence of hallux flexion deformity following free vascularized fibula flap transplantation. The study found a 100% incidence of this deformity. Wu et al. attributed the deformity to ischemic contracture of the FHL, which was caused by injury to the peroneal artery during the surgical procedure [14]. On the other hand, Daniels et al. found an 18% incidence of clawing of the great toe following the same procedure [15]. Sieg et al. explored donor site morbidity following free fibula transfer, revealing a 27% incidence of hammer and claw toes, along with deficits in the dorsal extension of the hallux [8].

The information gained by clinico-radiological assessment at four years of follow-up suggests that weakness of the EHL is a possible factor for the appearance of hallux deformities following fibula transposition surgery. Further detailed study in this regard is suggested. 

Management of hallux IPJ flexion contractures includes both conservative measures and surgical interventions. In our case, conservative measures, including physiotherapy and the use of a hallux extension splint, have been used. However, the possibility of surgical intervention, such as IPJ fusion, has been discussed. The decision regarding the timing and nature of the surgical intervention is influenced by various factors, including patient preferences, functional impairment, and disease progression.

## Conclusions

In conclusion, this case report highlights a unique instance of hallux IPJ flexion contracture in a 10-year-old patient with NF1 following fibula transposition surgery. The loss of the proximal fibular attachment of the EHL is postulated to have led to EHL weakness and an unopposed pull by FHL. Thorough diagnostic assessments, including imaging and NCS, were crucial in ruling out other etiologies and supporting this hypothesis. This case underscores the importance of considering prior surgical interventions in the differential diagnosis of toe flexion contractures and highlights the need for a multidisciplinary approach in managing such complex cases. Currently, conservative management with physiotherapy and a hallux extension splint is being pursued, while surgical options remain a consideration for the future. This report adds to the limited literature on the musculoskeletal complications associated with fibula harvesting and NF1, providing valuable insights for surgeons dealing with similar presentations.
